# **Cellular delivery of relaxin-2 mRNA as a potential treatment for kidney fibrosis**

**DOI:** 10.1016/j.mtbio.2023.100716

**Published:** 2023-06-27

**Authors:** Chenguang Ding, Bo Wang, Xiang Feng Lai, Yingcong Guo, Greg Tesch, Xiaoming Ding, Jin Zheng, PuXun Tian, Sharon Ricardo, Hsin-Hui Shen, Wujun Xue

**Affiliations:** a Department of Kidney Transplantation, Nephropathy Hospital, The First Affiliated Hospital of Xi'an Jiaotong University, Xi'an, Shaanxi, 710061, PR China; b Department of Materials Science and Engineering, Monash University, Clayton, Victoria, Australia; c Department of Nephrology and Monash University Department of Medicine, Monash Medical Centre, Clayton, Victoria, Australia; d Monash Biomedicine Discovery Institute, Department of Pharmacology, Monash University, Clayton, Victoria, Australia; e Institute of Organ Transplantation, Xi'an Jiaotong University, Xi'an, 710061, China

**Keywords:** Kidney injury, mRNA, Cubosome

## Abstract

Renal fibrosis is a pathological feature of chronic kidney disease and its progression correlates with kidney function impairment. Since there are currently no specific therapies for renal fibrosis, we explored whether inducing local production of the anti-fibrotic molecule relaxin-2 in kidney cells has potential as a strategy for suppressing the development of renal fibrosis. Our study examined whether delivery of relaxin-2 mRNA to kidney cells in vitro and in vivo could inhibit mechanisms leading to renal fibrosis. Transfecting relaxin-2 mRNA into cultured kidney cells inhibited fibrotic responses to TGF-β1 in an autocrine or paracrine manner by reducing fibrotic gene expression in kidney tubules, and reducing proliferation in kidney fibroblasts and mesangial cells. Similarly, cubosomes assisted delivery of relaxin-2 mRNA to mouse kidneys alleviated the fibrosis and inflammation associated with renal injury following unilateral ureter obstruction (UUO). Therefore, relaxin-2 mRNA exhibits potential as a novel therapy for inhibiting fibrosis and inflammation in chronic kidney disease.

## Graphical abstract

**Figure:**
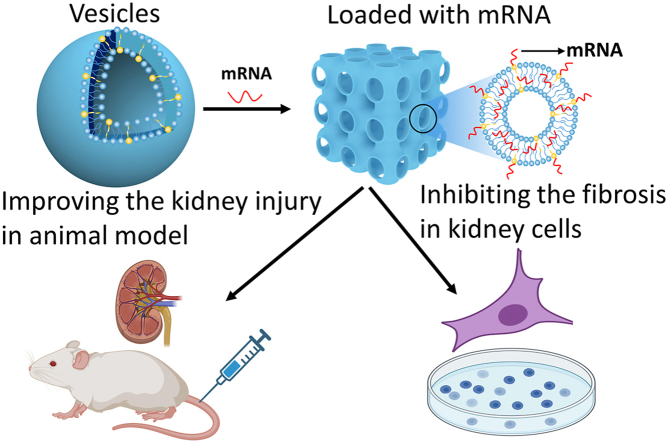

## Abbreviation list

α-SMA: alpha-smooth muscle actin
CKD: Chronic Kidney Disease
Col: Collagen
DN: Diabetic Nephropathy
TNF alpha: Tumour Necrosis Factor alpha
UUO: Unilateral Ureteral Obstruction

## 1 Introduction

**Chronic kidney disease.** The growing prevalence of chronic kidney disease (CKD) is a major health concern and can be caused by a variety of factors including obesity, diabetes and hypertension. Current therapies have a limited ability to prevent chronic kidney inflammation and fibrosis and halt the progression of CKD. Consequently, many CKD patients will develop end-stage kidney disease (ESKD) and require dialysis or kidney transplantation to survive. Therefore, novel strategies are required to specifically target chronic inflammation, and its ability to promote fibrosis, in patients with CKD.

Chronic inflammation causes injury and fibrosis in kidney glomeruli and the tubulointerstitium, leading to progressive CKD and renal function impairment. Complex mechanisms underly the development of kidney inflammation, which includes kidney accumulation of infiltrating leukocytes and increased levels of inflammatory cytokines [1].

**Relaxin-2 can suppress chronic kidney disease.** Recent studies have shown that the insulin-related endogenous peptide hormone relaxin-2 can alleviate CKD through regulating cell signalling pathways via cognate G protein-coupled receptor, relaxin family peptide receptor-1 [RXFP1] [2] and the glucocorticoid receptor [3]. Relaxin-2 greatly reduces the deposition of collagens in experimental models of acute and chronic kidney injury [4]. Recently, the therapeutic efficacy of recombinant protein of relaxin-2 (serelaxin) has been assessed in clinical trials of patients with heart failure or liver cirrhosis, with and without renal dysfunction [5,6]. These studies showed limited or inconclusive benefits in patients, suggesting that administration of recombinant relaxin-2 protein might not act as effectively as expected in clinical settings. Therefore, it may be a more optimal strategy to produce natural relaxin-2 directly in patients in their affected organs.

**mRNA based therapeutics.** The use of mRNA encoding a therapeutic protein has emerged as a viable strategy for disease treatment in areas such as therapeutic vaccines and immunotherapies for cancer [[7], [8], [9]]. Unlike conventional therapies involving synthesized inhibitors, activators, or recombinant proteins, nucleic-acid (mRNA) based strategies make use of the translational machinery of the mammalian cells to produce the therapeutic protein within the patient. mRNA-based therapeutics have two major advantages. Firstly, mRNA therapies are very precise and lack the off-target effects of some drugs, allowing the possibility of personalized treatments. Secondly, recent advancements in RNA technology make it possible to rapidly develop mRNA therapies which are cost effective and can be delivered safely, reliably and efficiently to yield their desired benefits. The successful application of mRNA-based COVID-19 vaccines in 2021 has proven the feasibility and effectiveness of mRNA therapies on a large scale. Therefore, utilizing mRNA to induce cells to create therapeutic proteins has immense potential for improving patient outcomes.

One of the hurdles in mRNA therapy is to find an efficient method for delivering mRNA to target cells in diseased tissues. This is complicated because mRNAs can be rapidly and easily degraded within and outside cells. Furthermore, optimal benefit usually requires that therapeutic mRNA is selectively delivered to the injured organ. The use of nanoparticles as mRNA carriers is a potential way to bridge this gap [10]. Cubic lyotropic liquid crystalline nanoparticles, known as cubosomes are considered to be the likely ‘next generation’ of lipid-based nanocarriers [11,12] due to their nanoscale size, biocompatible constituents, and high loading potential for hydrophobic, hydrophilic, and amphiphilic agents [13,14]. Thus, cubosomes have significant potential for delivering mRNA therapies in clinical applications.

In the current study, we created a chemically modified mRNA to produce relaxin-2 protein, and also developed a novel lipid-based nanoparticle (cubosomes) to deliver this mRNA for in vitro and in vivo applications. Importantly, the relaxin-2 protein translated from this mRNA was able to exert anti-fibrotic and anti-inflammatory functions as predicted when delivered by cubosomes. Therefore, our study demonstrates that cubosomes-delivery of relaxin-2 mRNA is a potential new therapy for treating CKD.

### 1.1 Materials and methods

**Cubosome formulations.** Phytantriol (98.0%, 3,7,11,15-tetramethylhexadecane-1,2,3-triol), Pluronic F127 and 1,2-dioleoyl-3-trimethylammonium-propane (chloride salt) (18:1 ​TAP (DOTAP)) were purchased from Sigma-Aldrich. Ultrapure water 18.2 ​MΩ ​cm (Milli-Q) was used as necessary. All chemicals were used as received without further purification.

Empty nanoparticles (defined as vesicles) were obtained by adding phytantriol (20 ​mg), DOTAP (3 ​mg) and F127 (2.3 ​mg) to a glass vial. The mixtures were then mixed thoroughly in chloroform and subjected to nitrogen gas drying to remove the chloroform. Afterward, the vial was placed in a desiccator for further drying under vacuum at room temperature overnight. After drying, Milli-Q water was added to the mixtures and subjected to sonication for 5 ​min (5 ​s pulses interrupted by 5 ​s) in an ice bath using an automated probe sonicator at 50% of maximum power (125-Watt, 20 ​kHz). Cubosomes (Vesicles ​+ ​mRNA) was prepared by mixing 20% w/w mRNA to vesicles and stirred for 10 ​min at room temperature. Rhodamine -labeled vesicles/cubosomes were prepared by adding Rhodamine to the mixtures prior to chloroform drying.

**Dynamic light scattering.** The samples’ diameter, size distribution, polydispersity index and zeta potential were measured on Malvern Zetasizer nanoZS at a concentration of 0.1 mg/ml in water. Samples in DTS 1070 folded capillary cells were stabilized at 37 ​°C and the results were recorded in triplicate.

**Small-angle X-ray scattering.** Bruker N8 Horizon (Software Diffrac. Suite V7.3.1, Monash X-ray Platform, Monash University, Australia) was used to allow irradiation of samples at room temperature within a sealed X-ray tube HV generator (Kα radiation from Cu-anode, wavelength λ ​= ​1.5406 ​Å) at 50 ​keV and 1000 ​μA. The investigated Q-range was from 0.007 to 0.387 ​Å^−1^ (scattering vector Q ​= ​[4π sin (θ/2)]/λ, where θ is the scattering angle). Samples were loaded into a 1 ​mm quartz capillary with glassy carbon as the reference for determining sample transmission. The 2D scattering pattern was then recorded by VÅNTEC-500 Detector for 2 ​h. The image was then integrated into the 1D scattering function I(Q) using DIFFRAC. SAXS software (Version 1.0, Monash X-ray Platform, Monash University, Australia). A silver behenate standard with a d spacing value of 58.38 ​Å was used for calibration. The lattice parameter a was calculated as: a ​= ​d_hkl_ $\sqrt{\left(h^{2}+k^{2}{+l}^{2}\right)}$, where the lattice spacing d_hkl_ ​= ​2π/Q, h, k and l are the miller indices.

**Cryogenic transmission electron microscopy.** To prepare the samples, the humidity in the room was kept close to 80%, and the ambient temperature at 22 ​°C. Briefly, 3 ​μl aliquots of the sample were added onto a 300-mesh copper grid, which was coated with perforated carbon film (Lacey carbon film: ProSciTech #GSCU300FL-50, QLD, Australia). 5 s later, the grid was blotted using Whatman 541 filter paper for 2 ​s. The grid was then quickly plunged into liquid ethane cooled by liquid nitrogen. The samples were examined using a Gatan 626 cryoholder (Gatan, Pleasanton, CA, USA) and Tecnai 12 Transmission Electron Microscope (FEI, Eindhoven, The Netherlands) at an operating voltage of 120 ​KV. At all times, low-dose procedures were followed, using an electron dose of 8–10 electrons/Å2 for all imaging. Images were recorded by FEI Eagle 4 ​k ×4 ​k CCD camera at a range of magnifications using AnalySIS v3.2 camera control software (Olympus).

### 1.2 In vitro studies - cell culture

The rat kidney tubular epithelial cell line (NRK52E), rat kidney fibroblast cells (NRK49F) and human embryonic kidney 293 ​cells (HEK293) were obtained from the American Tissue Culture Collection (Rockville, MD) and maintained in Dulbecco's modified Eagle medium containing 10% serum as previously described [15,16]. Mesangial cells and proximal tubular epithelial cells were isolated from the kidneys of C57BL/6 mice [17,18]. Gene expression in tubular epithelial and mesangial cell cultures was assessed after 3 days of stimulation with recombinant TGF-β1 (5 ​ng/ml) by quantitative real-time PCR.

The rat kidney fibroblast cells were obtained from the American Tissue Culture Collection (Rockville, MD) and maintained in Dulbecco's modified Eagle medium containing 10% serum and 5 ​mmol/l glucose.

For co-culture studies, kidney tubular NRK52E cells or kidney fibroblast NRK49F cells were cultured on transwell inserts in wells containing human embryonic kidney cells (HEK293) transfected with relaxin-2 mRNA. The medium in these wells was then replaced with fresh medium with or without TGF-β1, and the effects on NRK52E or NRK49F cells were examined after 72 ​h.

**Drugs and antibodies.** Recombinant human TGF-β1 protein, (R&D systems, Minneapolis, MN) and used at specified concentrations (5 ​ng/ml). Typically, 24 ​h after cells were seeded, culture media was replaced and cells were incubated for a further 1–5 days with TGF-β1, depending on experiments.

MTT assay. Media was discarded from cell cultures. 50 ​μL of serum-free media and 50 ​μL of MTT solution were added into each well. The plate was incubated at 37 ​°C for 3 ​h. After incubation, 150 ​μL of MTT solvent was added into each well. Read absorbance at OD ​= ​590 ​nm. Read plate within 1 ​h.

**RNA extraction and real-time PCR**. RNA was isolated from cells and tissues using a Zymo Quick RNA kit (Zymo Research, Irvine, CA 92614).

mRNA was reverse transcribed into cDNA using an Agilent AffinityScript kit (Agilent, Santa Clara, CA 95051). Gene expression was analyzed by real-time (RT)-PCR, using the TaqMan system based on real-time detection of accumulated fluorescence (ABI Prism 7500; Perkin-Elmer, Foster City, CA). Fluorescence for each cycle was quantitatively analyzed by an ABI Prism 7500 Sequence Detection System (Perkin-Elmer). To control for variation in the amount of DNA that was available for PCR in the different samples, gene expression of the target sequence was normalized in relation to the expression of an endogenous control, 18 ​S rRNA (18 ​S rRNA TaqMan Control Reagent kit, ABI Prism 7500; Perkin-Elmer). Details of primers and TaqMan probes (FAM) for these genes have been previously reported [15]. Each experiment was conducted in four to six replicates. Results were expressed relative to control (untreated) cells/tissue, which were arbitrarily assigned a value of 1.

**Transfection of mRNA. C**ells were seeded at 3 ​× ​10^4^ ​cells per well in 12-well plates. The following day, medium was replaced with OptiMEM (Invitrogen), and cells were transfected with relaxin-2 mRNA (1 ​ng) using Lipofectamine 2000 (Invitrogen).

In each case, non-translating (NT) mRNA controls were used at the same concentration. Cells were harvested 3–5 days post-transfection.

**ELISA** The expression levels of Relaxin-2 were determined in medium of cells using ELISA kits (R&D Systems, Inc., Minneapolis, MN, USA), according to the manufacturer's protocols.

**Animal models.** Unilateral ureter obstruction (UUO) was performed on C57BL/6 mice aged 7–8 weeks (20–25 ​g, n ​= ​7–8/group) [19], whereby the left ureter was visualized via a flank incision and ligated using double tracks with 5.0 surgical silk. This procedure results in rapidly progressive tubulointerstitial fibrosis, which resembles lesions seen in CKD after 7 days.

mRNA-cubosomes treatment of UUO mice. Male C57BL/6 mice (20–25 ​g) were randomly allocated into groups receiving either non-translating (NT) mRNA-cubosomes control (10 ​ng mRNA/200 ​ng cubosomes) or relaxin-2 mRNA-cubosomes (10 ​ng mRNA/200 ​ng cubosomes) (n ​= ​7/group). Cubosome containing either NT mRNA or relaxin-2 mRNA was administered into mice by tail vein injection on days 1, 3 and 5 after UUO surgery. Seven days after UUO, mouse kidneys were collected for analysis of inflammation and fibrosis.

### 1.3 Histopathology analysis of UUO kidney samples

Formalin-fixed paraffin-embedded (FFPE) kidneys were sectioned at 4 ​μm and stained with hematoxylin and eosin (H&E) to assess changes in kidney structure.

### 1.4 Immunohistochemistry

For immunostaining, 4 ​μm FFPE sections were treated with 20% rabbit or goat serum for 30 ​min and then incubated with primary antibody in 3% BSA overnight at 4 ​°C. Sections were then placed in 0.6% hydrogen peroxide in methanol for 20 ​min to inactivate endogenous peroxidase. Bound primary antibodies were detected using a standard ABC-peroxidase system: avidin-biotin block, biotinylated secondary antibodies (rabbit anti-goat IgG, goat anti-rat IgG or goat anti-rabbit IgG) and ABC-peroxidase (Vector Laboratories, Burlingame, CA, USA). Sections were developed with 3,3-diaminobenzidine (Sigma) to produce a brown colour.

Statistical analysis. Values are shown as means ​± ​SEM, unless otherwise specified. GraphPad Prism (GraphPad Software) was used to analyze data by unpaired Student's t-test or by one-way analysis of variance followed by a Tukey's multiple comparison test. Pvalues <0.05 were considered significant.

## 2 Ethics statement

Animal studies were performed at the Monash Medical Centre Animal Facility (Clayton, Australia) and were approved by the Monash Medical Centre Animal Ethics Committee in accordance with the Australian Code of Practice for the Care and Use of Animals for Scientific Purposes, 7^th^edition (2004).

## 3 Results

### 3.1 Transfection of relaxin-2 mRNA into kidney cells produces relaxin-2 protein

To examine cellular uptake and translation, relaxin-2 mRNA was conjugated with Alexa 488 and transfected into HEK293 ​cells using lipofectamine 2000, which was then examined by fluorescence microscopy and ELISA analysis. The results show that relaxin-2 mRNA maintained its stability (Fig. 1B) and translational capability (Fig. 1A) for 96 ​h. The transfection efficiency with lipofectamine 2000 was approximately 100% (Fig. 1C). Similar results were found when using Hela cells (Fig. 1D and E).

**Fig. 1:**
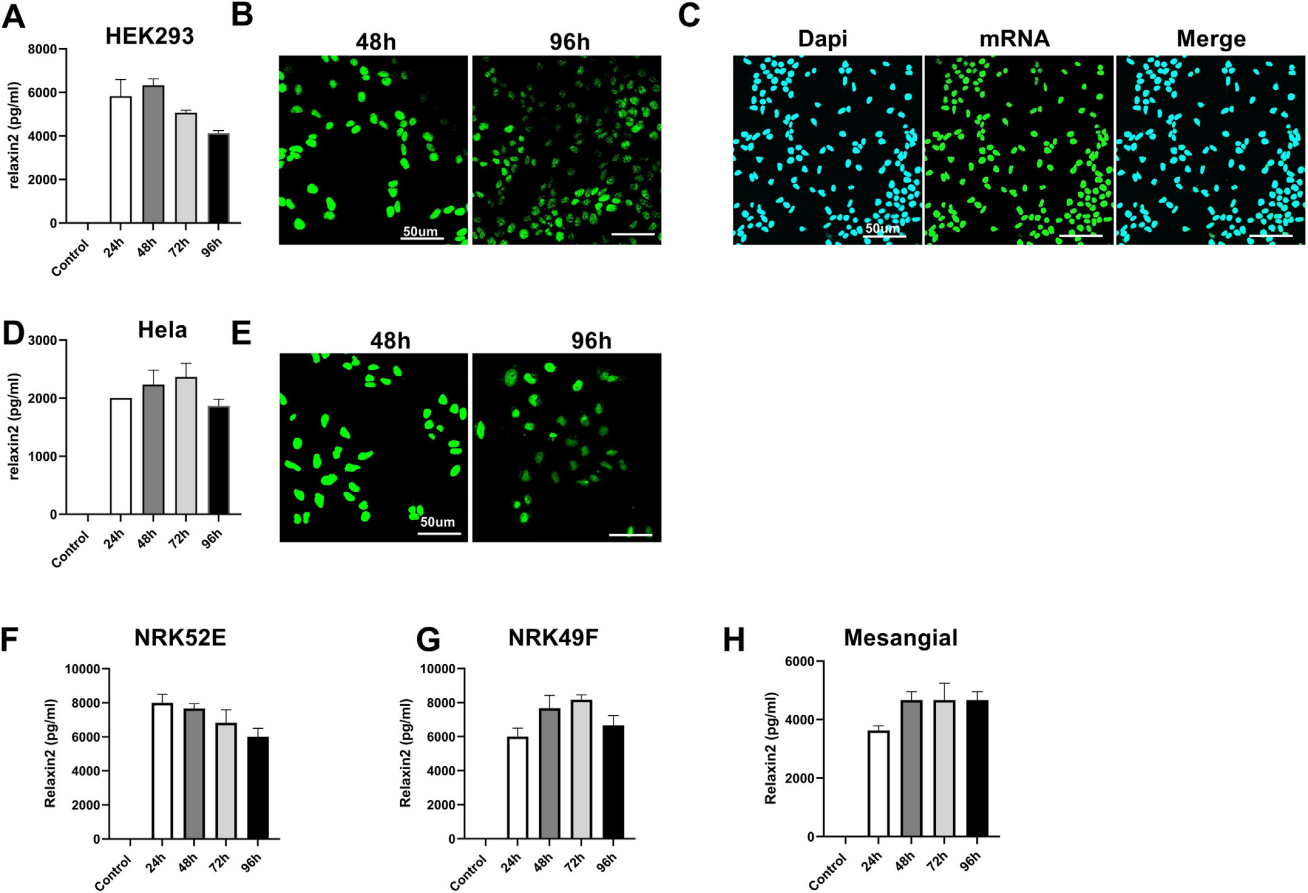
**Production of relaxin-2 protein in kidney cells by relaxin-2 mRNA.** Relaxin-2 ​mRNA maintained their translational capability (A) and stability (B) for 96 ​h in HEK293 ​cells with 100% transfection efficiency using lipofectamine2000 (C). Relaxin-2 ​mRNA also maintain stability and translational capability in Hela cells (D and E). Relaxin-2 ​mRNA maintained translational capability in kidney tubular cells (NRK52E) (F), kidney fibroblast cells (NRK49) (G) and mesangial cells (H). Results are expressed as the mean ​± ​SEM relative gene expression, ∗∗P ​< ​0.01, ∗P ​< ​0.05. N ​= ​3-5.

To verify that uptake of relaxin-2 mRNA could be translated throughout the kidney, mRNA was transfected into multiple kidney-derived cell types. ELISA results showed that relaxin-2 protein could be produced for at least 96 ​h after transfection of kidney tubular cells (NRK52E) (Fig. 1F), kidney fibroblast cells (NRK49) (Fig. 1G) and mesangial cells (Fig. 1H).

### 3.2 Overexpression of relaxin-2 in kidney cells directly regulates fibrotic mechanisms

**Fig. 2:**
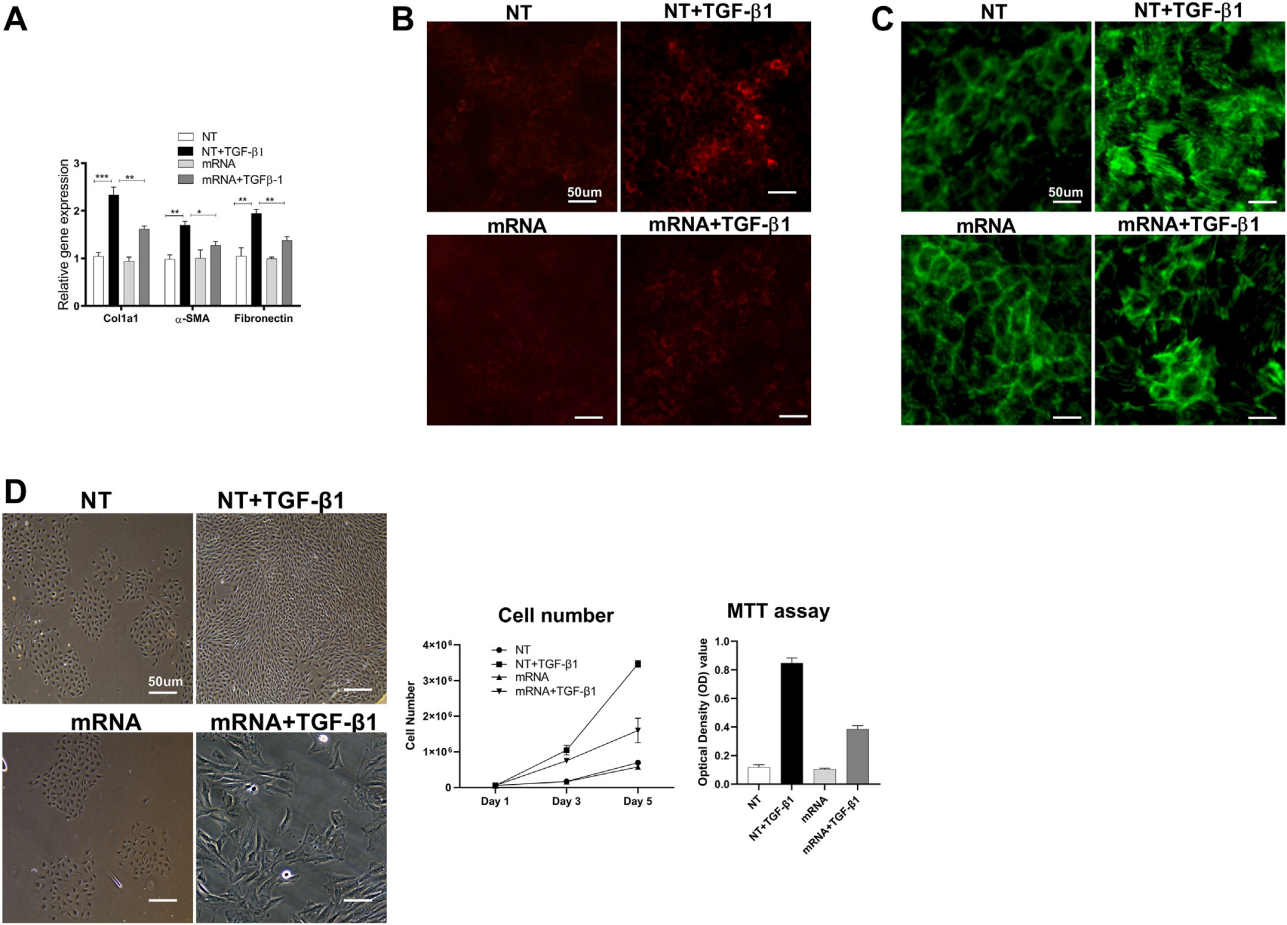
Relaxin-2 in kidney cells directly regulates fibrotic mechanisms. Relaxin-2 ​mRNA inhibited the overexpression of fibrotic markers in NRK52E cells by Q-PCR (A), consistent with the immunostaining of collagen1 (B). The re-organization of actin was alleviated by Relaxin-2 ​mRNA (C). Relaxin-2 ​mRNA inhibited the proliferation of kidney fibroblast (D), induced by TGF-β1. Results are expressed as the mean ​± ​SEM relative gene expression, ∗∗P ​< ​0.01, ∗P ​< ​0.05. N ​= ​3-5.

To verify that cellular uptake and transcription of relaxin-2 mRNA could regulate fibrosis in kidney cells, we transfected cultured kidney tubular cells (NRK52E) and kidney fibroblasts (NRK49F) with the relaxin-2 mRNA or non-translational mRNA (NT) for 72 ​h in the presence or absence of TGF-β1. Stimulation of kidney tubular cells with TGF-β1 elevated gene expression of collagen 1, alpha-smooth muscle actin (SMA) and fibronectin, which were inhibited in cells receiving relaxin-2 mRNA (Fig. 2A). Similarly, immunofluorescence staining demonstrated that TGF-β1 induces production of collagen 1 protein in tubular cells, which could be inhibited by treatment with relaxin-2 mRNA (Fig. 2B). The reorganization of actin cytoskeleton could also be partially restored by relaxin-2 mRNA (Fig. 2C). In addition, stimulation with TGF-β1 for 5 days induced kidney fibroblast proliferation, which was inhibited by relaxin-2 mRNA treatment (Fig. 2D), consistent with previous discovery about relaxin-2 regulation on fibroblast proliferation [20].

**Fig. 3:**
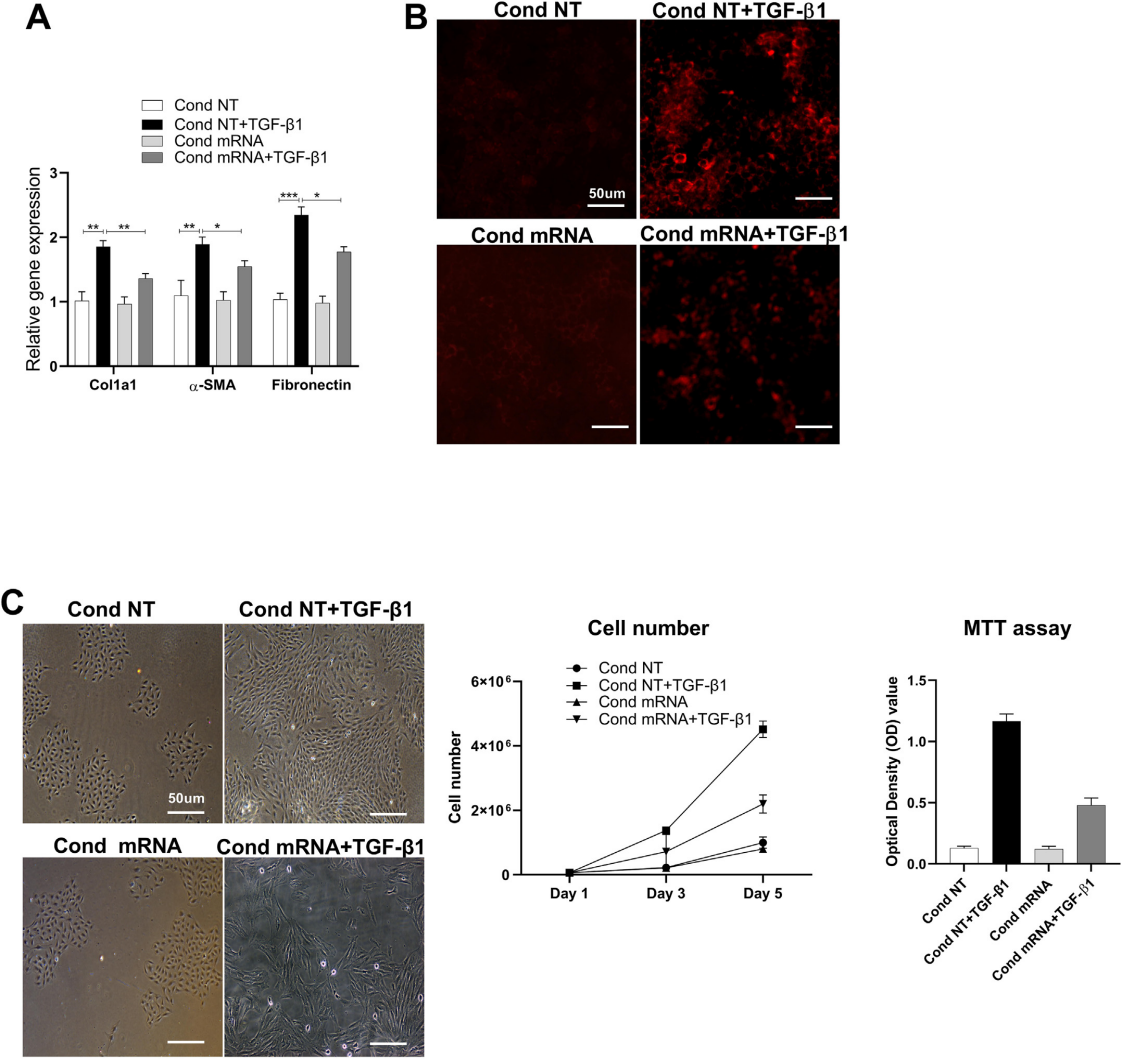
Paracrine effects of relaxin-2 protein on adjacent kidney cells. In the transwell co-culture system, the relaxin-2 produced by HEK293 ​cells in conditioned medium (cond), inhibited the overexpression of fibrotic markers (A and B), proliferation of kidney fibroblast cells (C), following TGF-β1 stimulation. Results are expressed as the mean ​± ​SEM relative gene expression, ∗∗P ​< ​0.01, ∗P ​< ​0.05. N ​= ​3-5.

To demonstrate the paracrine effects of relaxin-2 protein on adjacent kidney cells, we used a transwell co-culture system. Kidney tubular NRK52E cells or kidney fibroblast NRK49F cells, were cultured on transwell inserts in wells containing HEK293 ​cells previously transfected with relaxin-2 mRNA, allowing secreted molecules from transfected cells to interact with the kidney cells. We observed that production of relaxin-2 by HEK293 ​cells (in conditioned medium) could inhibit the expression of fibrotic genes and collagen 1 protein in NRK52E kidney tubular cells (Fig. 3A–B) and cell proliferation in NRK49F kidney fibroblasts (Fig. 3C) at day 5 after TGF-β1 stimulation.

### 3.3 Characterization of relaxin-2 mRNA loaded into cubosomes

**Fig. 4:**
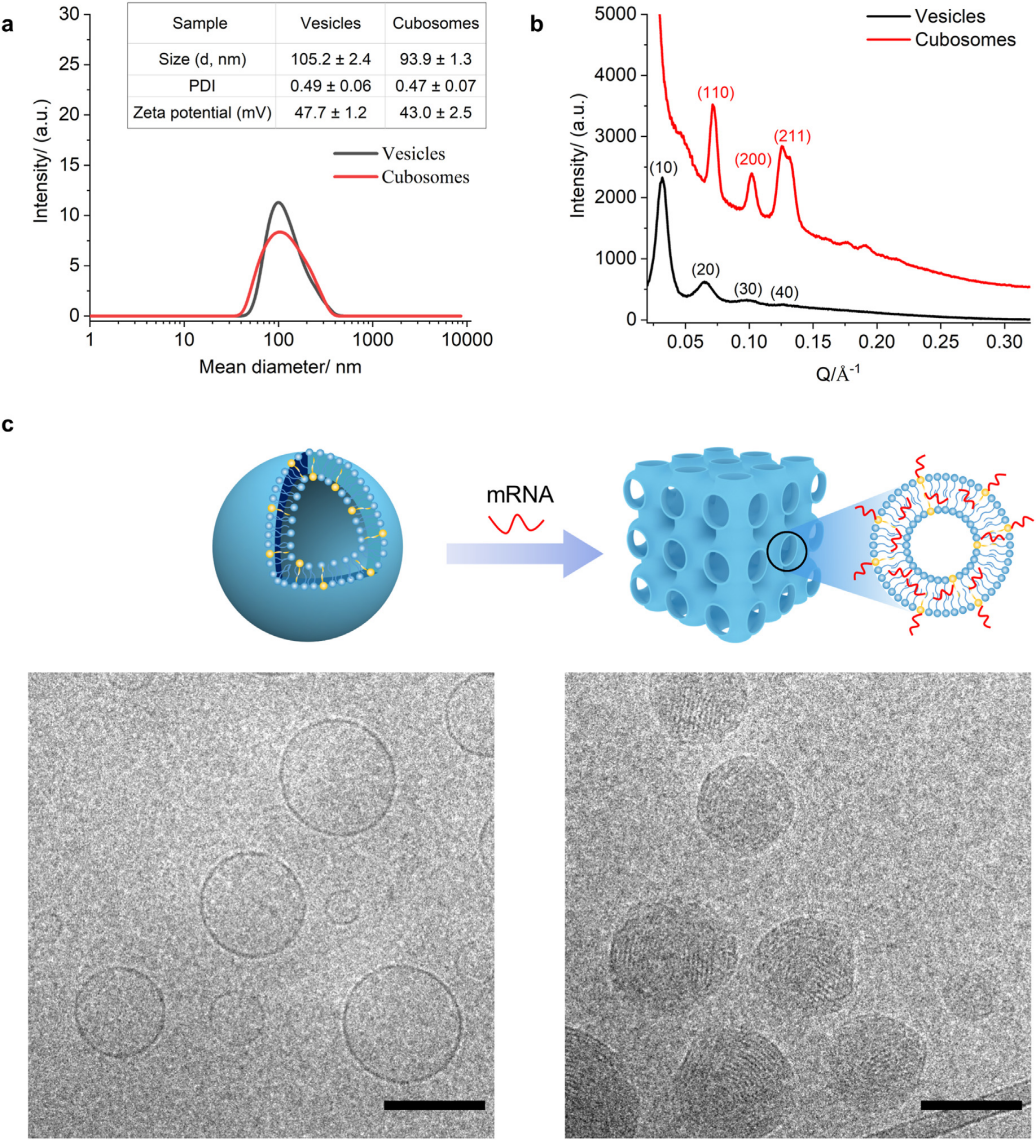
Characterization of vesicles and Relaxin-2 ​mRNA loaded cubosomes (A), Dynamic light scattering curves of vesicles and mRNA-loaded cubosomes. Results are summarized in the inserted table. Data are mean ​± ​s.d., based on values obtained from three independent experiments (n ​= ​3). d, diameter. PDI, polydispersity index. a. u., arbitrary unit. (B), Small angle X-ray scattering curves of vesicles (black curve) and mRNA-loaded cubosomes (red curve). a. u., arbitrary unit. (C), Cryogenic transmission electron microscopy of vesicles and mRNA-loaded cubosomes. Scale bar, 100 ​nm.

Dynamic light scattering revealed that vesicles without mRNA loading had a size and zeta potential of 105.2 ​± ​2.4 ​nm and 47.7 ​± ​1.2 ​mV, respectively (Fig. 4A). The addition of mRNA to the above vesicles decreased the size and zeta potential to 93.9 ​± ​1.3 ​nm and 43.0 ​± ​2.5 ​mV, respectively. Small angle X-ray scattering analysis was carried out to investigate the internal nanostructures of nanoparticles before and after encapsulation of mRNA. For unloaded nanoparticles, four well-resolved Bragg reflections correspond to the Miller indices of [10], [20], [30] and (40) (Fig. 4B), which indicated a multilayer lamellar phase (L_α_) with large d-spacing (a ​= ​19.6 ​nm) in the low scattering vector region. Encapsulation with mRNA transited the structure to Im*3m* cubic space group, as indicated by the three well-resolved Bragg reflections corresponding to the Miller indices of (110), (200) and (211) (Fig. 4B), and also decreased the lattice parameter to a ​= ​12.3 ​nm. This suggests that the presence of hydrophilic mRNA led to the shrinkage of the water channels. Cryogenic transmission electron microscopy clearly showed that structure of the vesicles, while the encapsulation of mRNA induces highly ordered internal nanostructures in these particles (Fig. 4C).

### 3.4 Cellular update of the cubosomes loaded with mRNA

**Fig. 5:**
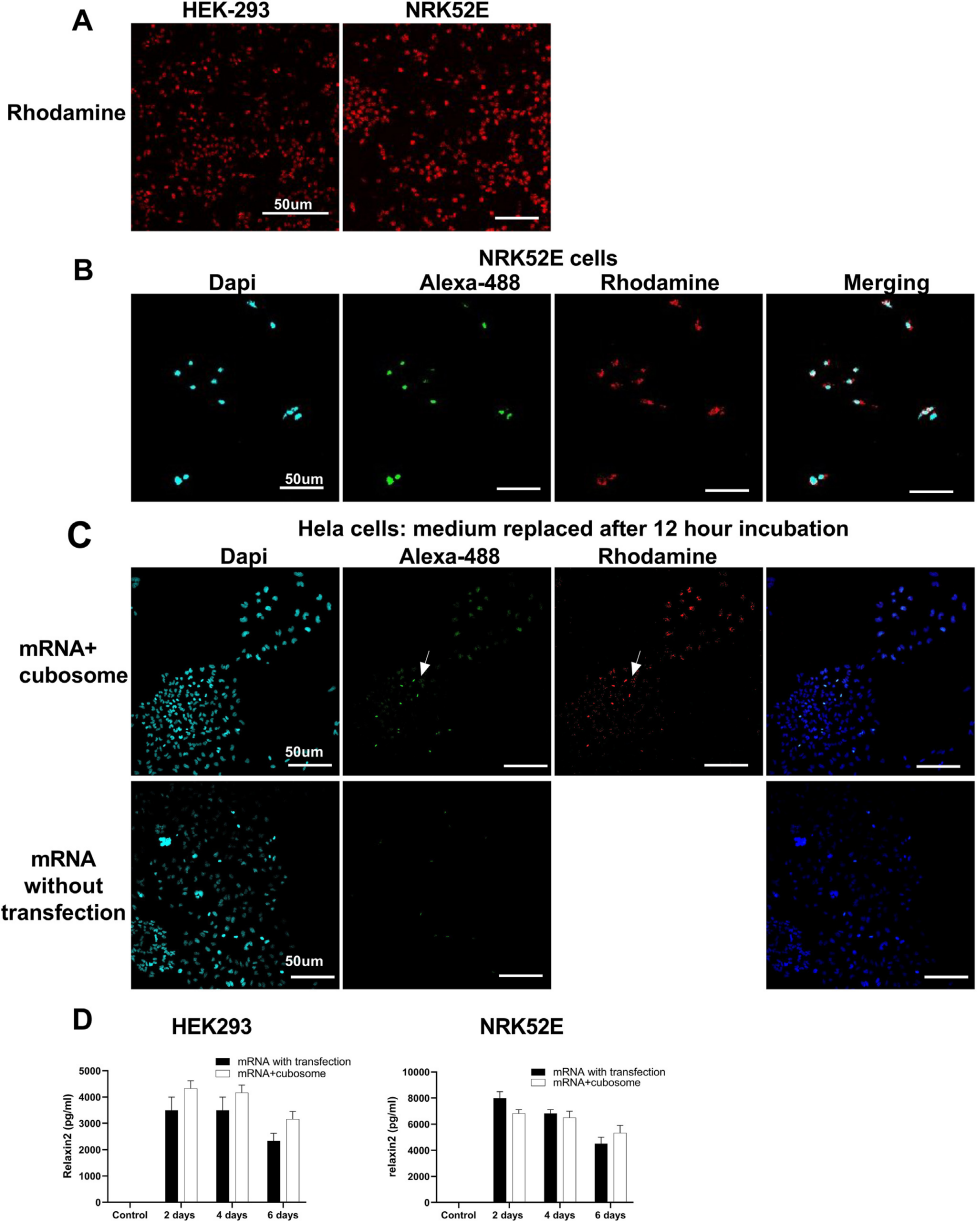
Cellular uptake of the cubosomes. Microscopy showed uptake of cubosomes by HEK293 ​cells and NRK52E cells using fluorescence detection of rhodamine (A). Merged channels of cubosomes (Rhodamine), mRNA (Alexa-488) and nuclear (Dapi) in NRK52E cells (B). Hela cells were washed after 12 ​h after the addition of cubosomes complex loaded with mRNA or mRNA alone without transfection. After 2 days incubation, the expression efficiency of cubosomes and mRNA were much higher than mRNA alone (C). An additional experiment showed significant levels of secreted relaxin-2 were identified at 2 days post transfection and were similar in cells receiving mRNA with lipofectamine transfection or cubosomes with mRNA (D).

To examine cellular uptake of the cubosomes-mRNA complex, we labeled the cubosomes with rhodamine. Microscopy showed uptake of cubosomes by HEK293 ​cells and NRK52E cells using fluorescence detection of rhodamine (Fig. 5A). Fig. 5B showed that the merged channels of cubosomes (Rhodamine), mRNA (Alexa-488) and nuclear (Dapi) in NRK52E cells.

To compare the uptake efficiency of cubosomes loaded with relaxin-2 mRNA with relaxin-2 mRNA without transfection, Hela cells were washed after 12 ​h of incubation and analyzed at 48 ​h. Analysis showed that the expression efficiency of cubosomes-delivered mRNA was much higher than mRNA without transfection (Fig. 5C).

We also demonstrated that HEK293 ​cells and NRK52E cells were able to translate the relaxin-2 mRNA after cubosomes delivery by measuring secretion of relaxin-2 protein. Significant levels of secreted relaxin-2 were identified at 2 days post transfection and were similar in cells receiving mRNA with lipofectamine 2000 transfection or cubosomes with mRNA (Fig. 5D), with levels gradually declining between days 4 and 6. Kidney tubular cells (NRK52E) secreted more relaxin-2 protein than embryonic kidney cells (HEK293).

### 3.5 Delivery of cubosomes-loaded mRNA into mouse organs

**Fig. 6:**
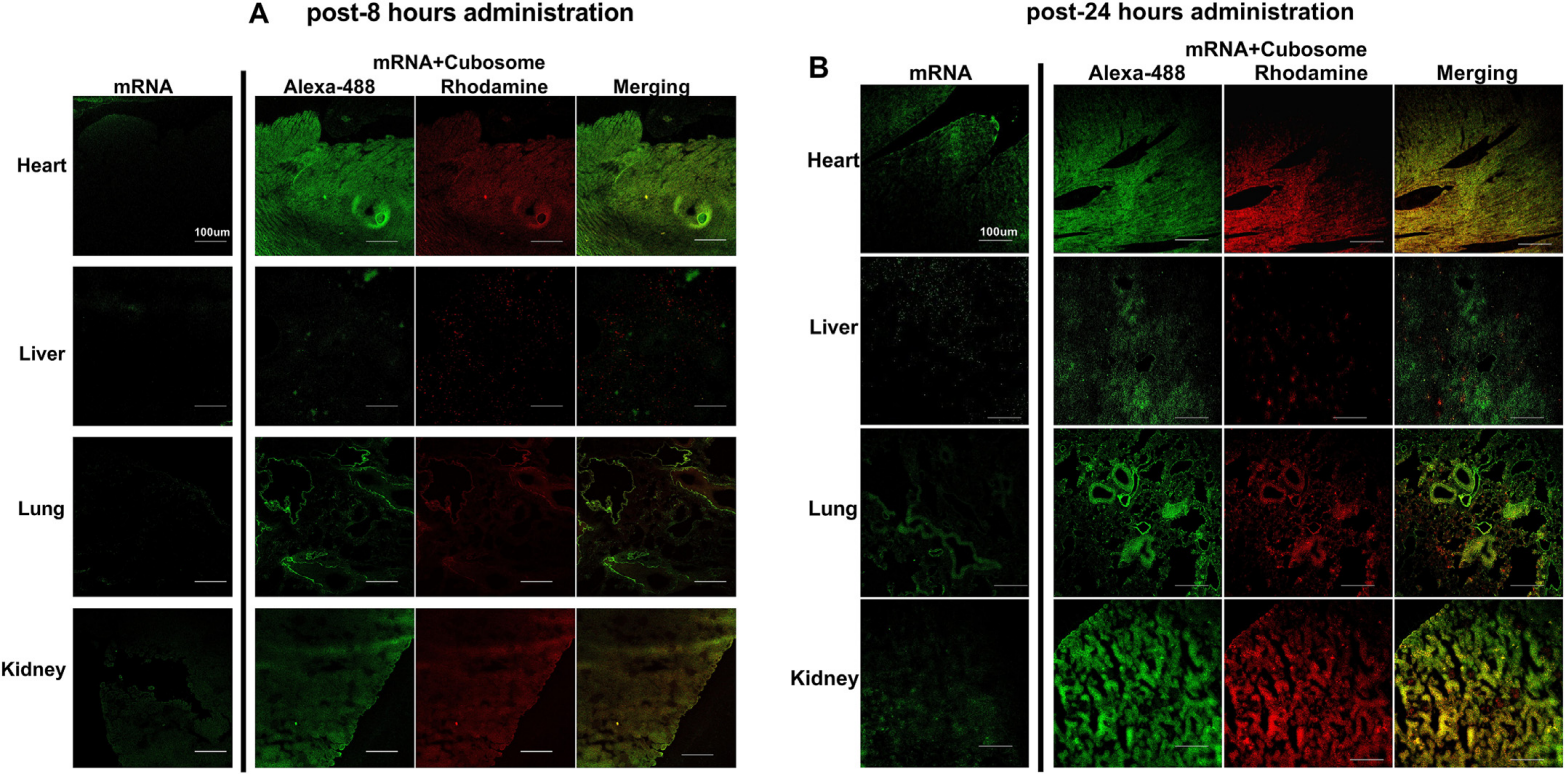

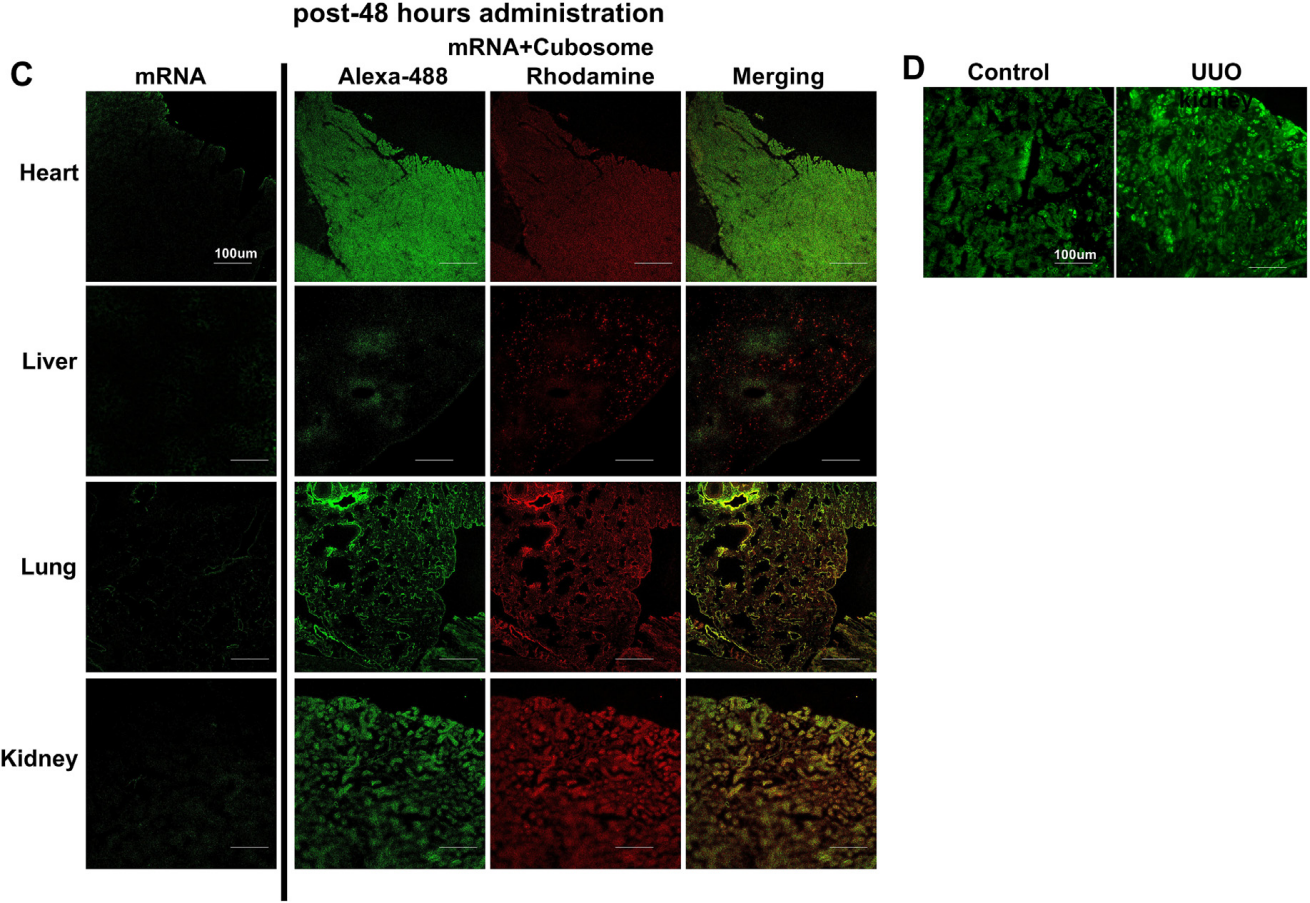
Delivery of Cubosome-loaded mRNA into mouse organs Cubosome containing Alexa-488-labeled relaxin-2 mRNA was administered into a C57BL/6 mouse. Eight hours after injection, a fluorescence signal identified uptake of cubosomes and mRNA in kidney, liver, heart and lung tissues (A). This uptake was even greater at 24 ​h (B) and 48 ​h (C). In addition, the Alexa-488-conjugated relaxin-2 mRNA was observed at day 7 after giving 3 doses of cubosomes-mRNA at 48-h intervals to either normal mice or UUO mice (D).

To examine the distribution pattern of mRNA in vivo, we injected cubosomes containing Alexa-488-labeled relaxin-2 mRNA into the tail vein of a C57BL6/J mouse. Eight hours after administration, we identified a fluorescence signal indicating uptake of cubosomes and mRNA in heart, kidney, liver and lung tissues (Fig. 6A). Fluorescence imaging indicated an even greater uptake of cubosomes and mRNA in each of these organs at 24 ​h (Fig.s. 6B) and 48 ​h (Fig. 6C). In addition, giving 3 doses of cubosomes-mRNA at 48-h intervals (days 1, 3 and 5 after UUO) resulted in significant uptake of Alexa-488-conjugated relaxin-2 mRNA in UUO kidneys at day 7, which was comparable to equivalent dosing in normal mouse kidneys (Fig. 6D). Haemotoxylin and Eosin staining was used to demonstrate the structures of specific mouse organs used in this study (Supplementary data 1).

Administration of cubosomes-loaded relaxin-2 mRNA reduces inflammation and fibrosis in mouse UUO kidneys.

We examined the ability of cubosomes delivered relaxin-2 mRNA to reduce inflammation and fibrosis in mouse UUO kidneys by administering cubosomes-loaded relaxin-2 mRNA to mice on days 1, 3 and 5 after UUO surgery.

**Fig. 7:**
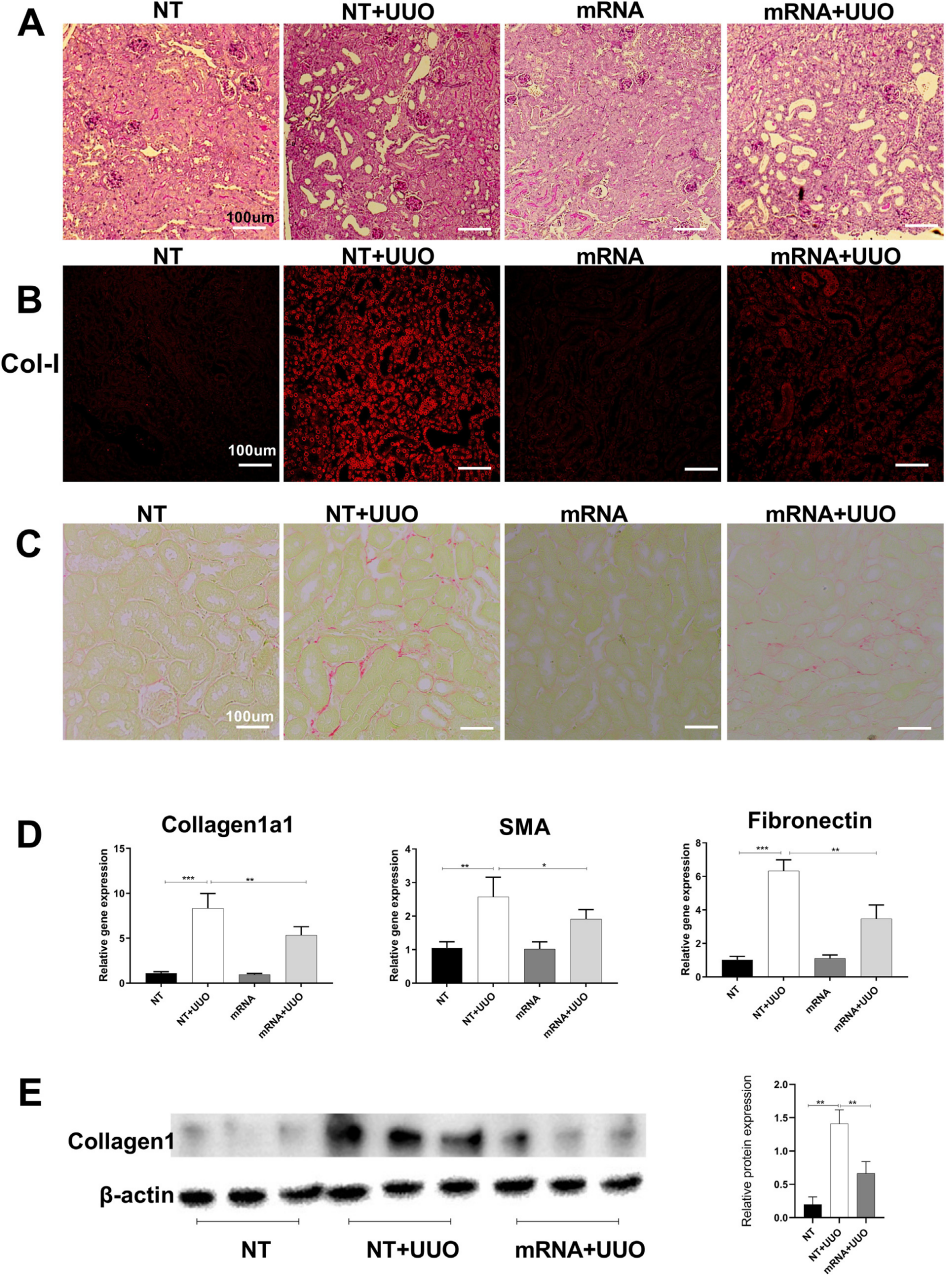
Administration of Cubosome-relaxin-2 mRNA complex reduces fibrosis in mouse UUO kidneys At day 7 after UUO surgery, histological staining showed that mice receiving no treatment, non-translational (NT) mRNA or relaxin-2 mRNA had similar injury to kidney tubules, which included dilation and atrophy of cortical tubules and tubule cast formation (A). Immunostaining indicated the up-regulation of collagen in UUO kidneys was alleviated by the administration of mRNA (B), consistent with PSR staining (C). Fibrotic makers upregulated in UUO kidney receiving relaxin-2 mRNA were significantly alleviated (D and E).

At day 7 after UUO surgery, histological staining showed that mice receiving non-translational (NT) mRNA or relaxin-2 mRNA had similar injury to kidney tubules, which included dilation and atrophy of cortical tubules and tubule cast formation **(**Fig. 7A). This indicated that the kidney injury caused by UUO surgery was consistent across all experimental mice.

In an analysis of kidney interstitial fibrosis, immunostaining identified a marked increase of kidney collagen 1 deposition in UUO mice receiving NT mRNA, which was suppressed in UUO mice treated with relaxin-2 mRNA (Fig. 7B). This observation is consistent with the findings of picrosirius red staining (PSR) (Fig. 7C).

Cubosome delivery of relaxin-2 mRNA had no effect on normal mouse kidney expression of fibrotic genes compared to non-translational (NT) mRNA. In contrast, mice with UUO that received NT mRNA had significantly increased kidney gene expression of collagen I, fibronectin and α-smooth muscle actin (SMA) (Fig. 7D), which was reduced in mice with that received cubosomes with relaxin-2 mRNA (Fig. 7D). Western blot analysis confirmed a reduction of collagen protein in mice receiving relaxin-2 mRNA (Fig. 7E).

**Fig. 8:**
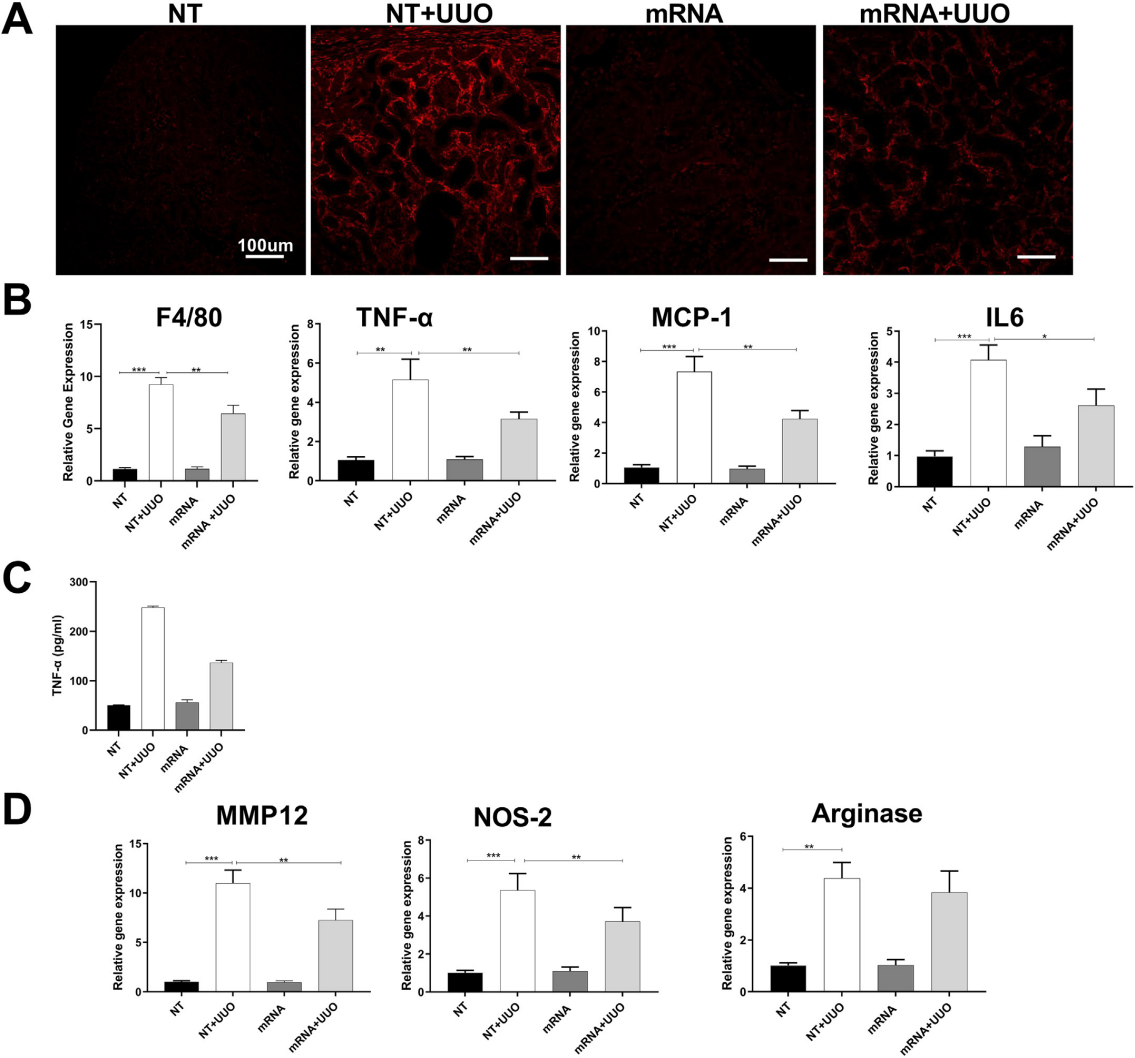
Cubosome-relaxin-2 mRNA complex alleviated inflammation in mouse UUO kidneys The up-regulation of F4/80+ macrophages in the kidneys of mice with UUO was significantly reduced by treatment with relaxin-2 mRNA (A) by immunostaining. PCR analysis identified the overexpression of inflammatory genes (TNF-α, MCP-1, IL-6) in mouse UUO kidneys was reduced in mice receiving relaxin-2 mRNA (B). Elisa of TNF-alpha was performed (C). M1 and M2 markers were analyzed (D). Results are expressed as the mean ​± ​SEM relative gene expression, ∗∗P ​< ​0.01, ∗P ​< ​0.05. N ​= ​3-5.

In an analysis of inflammation, immunostaining showed a marked increase in F4/80+ macrophages in the kidneys of mice with UUO, which was significantly reduced by treatment with relaxin-2 mRNA (Fig. 8A). This finding was further supported by PCR analysis that identified an increase in expression of the macrophage F4/80 gene (ADGRE1) and inflammatory genes (TNF-α, MCP-1, IL-6) in mouse UUO kidneys, which was substantially reduced in mice receiving relaxin-2 mRNA (Fig. 8B). Similarly, delivery of relaxin-2 mRNA was found to reduce the elevated levels of TNF-α protein in lysates of UUO kidneys (Fig. 8C) and the increased gene expression of macrophage M1 markers (Nos2, MMP-12), but not the M2 marker (Arginase 1), in UUO kidneys (Fig. 8D). These finding demonstrate that cubosomes delivery of relaxin-2 mRNA can reduce kidney inflammation and fibrosis in the UUO model of progressive renal fibrosis.

## 4 Discussion

This study has identified anti-fibrotic and anti-inflammatory roles for relaxin-2 mRNA in the progression of kidney injury. It has also established a novel cubosomes-delivery strategy for transferring relaxin-2 mRNA into rodent and human kidney cells, which was shown to have therapeutic benefits in a mouse model of progressive tubulointerstitial fibrosis. These findings highlight the clinical potential for kidney delivery of relaxin-2 mRNA to be used as strategy to treat ongoing fibrosis in patients with chronic kidney diseases.

Relaxin-2 is known to have anti-fibrotic and anti-inflammatory properties in diseases of the heart, liver and kidney [21,22]. However, recent clinical trials exploring the benefits of recombinant relaxin-2 have so far shown limited benefit. The administration of relaxin-2 protein has previously demonstrated controversial effects in some diseases, which has been proposed to be due to its short half-life in patients in recent clinical trials [[23], [24], [25]]. These unexpected outcomes may also be due to differing folding and post-translational modifications between native and recombinant relaxin-2 [[26], [27], [28]]. In contrast, the delivery of mRNA may produce a more prolonged and consistent availability of native relaxin-2 through local production, resulting in increased therapeutic benefits to patients associated with the known biological functions of relaxin-2.

In this study, we demonstrated the ability to deliver exogenous relaxin-2 mRNA into multiple kidney cells, thereby inducing them to produce the relaxin-2 protein and inhibit cellular responses to the profibrotic cytokine TGF-β1. This protection correlated with reduced levels of fibrotic markers and cell proliferation in kidney cells. The relaxin-2 regulation on fibroblast cell proliferation was demonstrated in cardiac fibrosis model [20]. Furthermore, our transwell studies showed that the relaxin-2 protein produced and secreted by our target kidney cells (e.g. proximal tubular epithelial cells) could exert anti-fibrotic paracrine effects on adjacent kidney cells of a different cell type (e.g. fibroblasts). Hence, introducing relaxin-2 mRNA into the most prevalent kidney cell type (tubular epithelial cells) can facilitate anti-fibrotic effects throughout the kidney.

The application of lipid-based nanoparticles for the delivery of biomolecules has attracted considerable attention due to the current interest in nucleotide-based therapeutics [29]. Our data has demonstrated that cubosomes loaded with mRNA can be endocytosed into kidney cells and exert mRNA translational function to produce relaxin-2 protein, suggesting the feasibility of cubosomes as a vehicle for nucleotide delivery. When administered systemically into mice, the cubosomes loaded with mRNA were distributed into multiple organs, with significant uptake and translation seen in both normal and injured kidneys. Tissue uptake of mRNA was seen at 8 ​h after administration, and was found to last at least 48 ​h. These findings indicate that cubosomes are effective carriers for delivering mRNA therapies into kidney cells during disease.

Other research also supports the concept of using nanoparticles to deliver mRNA-based therapies to treat diseases. One recent study demonstrated the feasibility in the treatment of ischemic neuronal death by introducing brain-derived neurotrophic factor mRNA using polyplex nanomicelles [30]. Similarly, another study showed that delivery of mRNA using synthetic lipid nanoparticles provided effective treatment of pulmonary lymphangioleiomyomatosis [31]. Therefore, it appears that nanoparticles may have potential for delivering mRNA therapies in a range of diseases. What remains to be determined are: how safe and reliable are these nanoparticles for patients; and, how selective do these nanoparticles need to be in delivering mRNA therapies to diseased organs in order to be clinically effective.

Our in vivo experiment validated relaxin-2 mRNA as a therapeutic strategy for kidney disease. In our mouse model of unilateral ureter obstruction (UUO), we demonstrated that kidney injury is associated with increased production of extracellular matrix proteins (collagen, fibronectin), accumulation of myofibroblasts (α-smooth muscle actin), and an inflammatory response involving macrophage infiltration (F4/80+ cells), an M1 macrophage phenotype and inflammatory cytokine production (TNF-α, IL-6, MCP-1), which is consistent with previous studies [32,33]. Treatment of UUO mice with cubosomes-loaded relaxin-2 mRNA reduced these fibrotic and inflammatory responses, thereby demonstrating a protective role for relaxin-2 mRNA in kidney injury. Therefore, we speculate that therapeutic administration of relaxin-2 mRNA has potential for suppressing the progression of chronic kidney disease (CKD) in patients, since many of the pathomechanisms seen in the UUO model also appear in patients with progressive CKD [34,35].

In conclusion, our study has established that delivery of relaxin-2 mRNA to kidneys cells is a novel treatment for suppressing the progression of kidney disease. This study also highlights the use of cubosomes nanoparticles as an effective means for transporting mRNA therapies into kidney cells during disease. These outcomes will provide direction for future studies that evaluate the clinical potential of relaxin-2 mRNA, using either cubosomes or other effective nanoparticle carriers to target diseased organs.

## Statement of author contributions

ChenGuang Ding designed experiments and performed some experiments, Bo Wang organized the whole project, designed experiments and performed some experiments, XiangFeng Lai designed and generated the cubosomes, Yingcong Guo helped design the experiments, Xiaoming Ding designed experiments and performed some experiments, Puxun Tian helped the organization of experiments and diagrams design, Jin Zheng helped the experiment design, Sharon Ricardo helped the administration of WISP1 antibody in animal experiments and carried out UUO model, Greg Tesch helped write part of the paper and provided suggestions in experimental design, Hsin-Hui Shen provided the laboratory space and relevant consumables, Wujun Xue provided ideas in this project and organized the team work.

## Funding statement

This work was supported by the mRNA Victoria Research Acceleration Fund (mVRAF); National Natural Science Foundation of China (10.13039/501100001809NSFC), Grant/Award Number: 82070768; Key research and development program of Shaanxi Province, Grant/Award Number: 2021 ​KW-53; Clinical Research Award of the First Affiliated Hospital of Xi'an Jiaotong University, Grant/Award Number: XJTU1AF-CRF-2019-008; Special Supportive Program for Organ Transplantation by COTDF, Grant/Award Number: 2019JYJH04; Transplantation Leadership Program” Research Support Project by COTDF, Grant/Award Number: YZLC-2021-008; Two chain integration project of 10.13039/501100011710Shaanxi Provincial Science and Technology Department, Grant/Award Number: 2021 ​L ​L-JB-06（3.5）

## Declaration of competing interest

There is no conflict of interest within the authors.

## Figures and Tables

**figs1:**
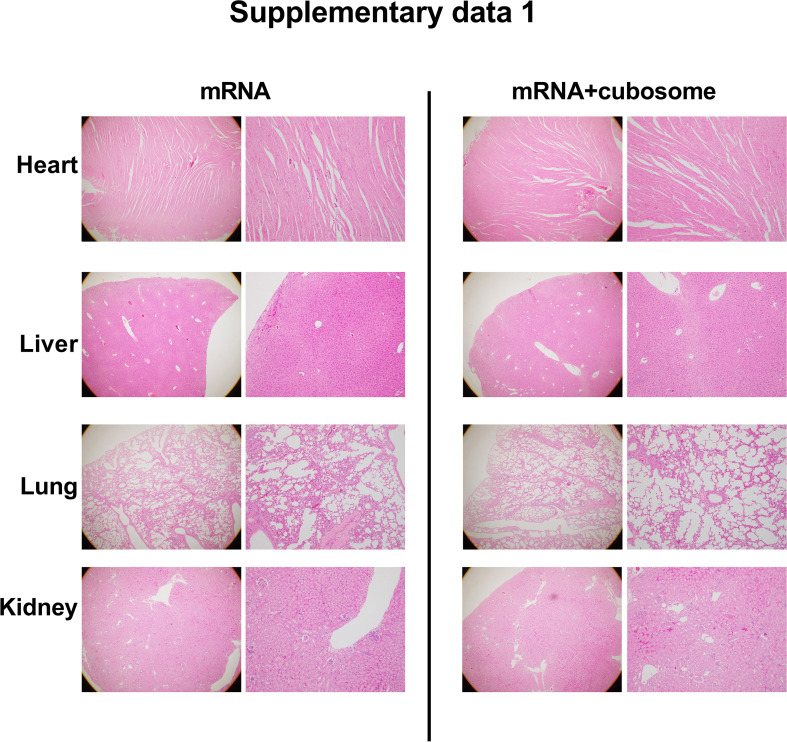


## Data Availability

Data will be made available on request.
